# *Cryptococcus gattii* molecular type VGII infection associated with lung disease in a goat

**DOI:** 10.1186/s12917-017-0950-6

**Published:** 2017-02-07

**Authors:** Evelin Catarine da Silva, Juliana Mariotti Guerra, Luciana Neves Torres, Alessandra Maria Dias Lacerda, Raquel Gonçalves Gomes, Danilo Marin Rodrigues, Rodrigo Albergaria Réssio, Priscilla Anne Melville, Camila Cecilia Martin, Fernando José Benesi, Lílian Rose Marques de Sá, Bruno Cogliati

**Affiliations:** 1 Departamento de Patologia, Serviço de Patologia Animal – Hospital Veterinário (HOVET), Faculdade de Medicina Veterinária e Zootecnia da Universidade de São Paulo (FMVZ-USP), Av. Prof. Dr. Orlando Marques de Paiva 87, Cidade Universitária, São Paulo, SP 05508-270 Brazil; 2 Instituto Adolfo Lutz (IAL) - Centro de Patologia, Av. Dr. Arnaldo, 351 – 7o. Andar – Sala 705 - Pacaembú, São Paulo, SP 01246-000 Brazil; 3 Departamento de Medicina Veterinária Preventiva e Saúde Animal, Faculdade de Medicina Veterinária e Zootecnia da Universidade de São Paulo (FMVZ-USP), Av. Prof. Dr. Orlando Marques de Paiva 87, Cidade Universitária, São Paulo, SP 05508-270 Brazil; 4 Clínica de Bovinos e Pequenos Ruminantes – Hospital Veterinário (HOVET), Faculdade de Medicina Veterinária e Zootecnia da Universidade de São Paulo (FMVZ-USP), Av. Prof. Dr. Orlando Marques de Paiva 87, Cidade Universitária, São Paulo, SP 05508-900 Brazil

**Keywords:** Cryptococcosis, Pneumonia, Goat, Yeast, Public health

## Abstract

**Background:**

*Cryptococcus gattii*-induced cryptococcosis is an emerging infectious disease of humans and animals with worldwide distribution and public health importance due to its significant morbidity and mortality rate. The present study aimed to report a case of pulmonary infection by *C. gattii* molecular type VGII in State of São Paulo, Brazil.

**Case presentation:**

A 5-year-old goat showing intermittent dry cough, ruminal tympany, anorexia, fever, tachycardia and tachypnea was presented for necropsy at the Veterinary Hospital of the School of Veterinary Medicine and Animal Sciences, São Paulo University, São Paulo, Brazil. Postmortem examination revealed numerous 2.0–6.0 cm diameter yellow gelatinous pulmonary masses. Tissues were evaluated by a combination of pathological, mycological, and molecular diagnostic techniques. Microscopically, pneumonia granulomatous, multifocal to coalescing, moderate, with many intralesional carminophilic yeasts was observed. The immunohistochemistry and mycological culture confirmed *Cryptococcus* spp. Internal transcribed spacers and orotidine monophosphate pyrophosphorylase nucleotide differentiation demonstrated that the isolate corresponds to the *C. gattii* VGII molecular subtype.

**Conclusions:**

To our knowledge, this is the first report of a pulmonary infection in a goat linked to *C. gattii* molecular type VGII in Southeastern Brazil. Our findings emphasize the need for an active surveillance program for human and animal new infections to improve the current public health policies due to expansion of the epidemiological niche of this important microorganism.

## Background

Cryptococcosis is a life-threatening systemic mycosis affecting humans and a large variety of animals. The two main pathogenic species are *Cryptococcus neoformans* and *Cryptococcus gattii* [1]. *C. neoformans*, which has a worldwide distribution, is an important cause of morbidity and mortality in immunocompromised hosts (especially AIDS-patients) and commonly found in bird feces, nests, and tree bark. It is divided into the molecular types VN I and VN II (*var. Grubii*, serotype A), VN III (serotype AD), and VN IV (*var. Neoformans*, serotype D). Otherwise, *C. gattii*, which is found in leaves, tree bark, and fruits in tropical and subtropical areas as well as in eucalyptus flowers and usually infects immunocompetent hosts, is classified into serotypes B and C and into four molecular types: VGI, VGII, VGIII, and VGIV [2, 3].

There are reports of cryptococcosis in different domestic and wild animals. Dogs and cats become infected by inhalation of spores through the nasal cavity, and the infection spreads throughout the respiratory system and often reaches the nervous system. In horses, sheep, and goats, the lesions are restricted to the respiratory system; while in cattle, lesions are usually located in the mammary glands [4]. Wild animals such as koalas, snakes, ferrets, and porpoises can also be affected and show different clinical manifestations, which are restricted to lungs and central nervous system. In ferrets, *Cryptococcus* spp. can affect the gastrointestinal and respiratory systems such nasal cavity and eyes [5]. In contrast, there are few reports of cryptococcosis in birds because these animals are resistant to this fungal disease due to their high body temperature (41–43 °C) [6]. In humans, after inhalation, the disease progresses into the lungs, followed by involvement of the innate immune system, occurs tissue and macrophage invasion, and consequently, systemic dissemination [7].

In caprines, reports of pulmonary cryptococcosis are rare, and the main alterations observed include pleuropneumonia, nasal congestion, mucopurulent exudate, dyspnea, anorexia, and cachexia [8]. In Australia, skin granulomas on the head region and nasal cavity involvement were described in two goats [9, 10]. *C. neoformans* is rarely diagnosed as a pneumonia-causing agent [10], but according to Baró et al. [9], outbreaks in cattle and caprines have been reported. *C. neoformans* infection is occasionally reported in association with other diseases, such as caprine arthritis, encephalitis, pleuropneumonia, and neoplasms [11]. Thirteen autochthonous strains of *C. gattii* serotype B was recently reported to cause severe and disseminated pulmonary cryptococcosis in goats, as well as outbreaks of pneumonia, with cachexia and involvement of central nervous system in goats herds in Spain, however, molecular genotype was not identified [9]. In goats, *C. gattii* has been mainly associated with respiratory diseases, while *C. neoformans* mainly affects the central nervous system [12].

Cryptococcosis can be diagnosed by identification of oval and encapsulated budding organisms during cytologic examination of smears, histopathology, fungal isolation, culture and molecular tests [10] or in a pre-mortem diagnosis by cytology or antigen search in serum by latex agglutination [13]. Thus, the present study aimed to report a case of *C. gattii* molecular type VGII infection in a goat (*Capra aegagrus hircus*) as well as demonstrate and discuss the different diagnostic methods available for small ruminants.

## Case presentation

A 5-year-old mixed-breed goat (*Capra aegagrus hircus)* was necropsied at Veterinary Hospital of the School of Veterinary Medicine and Animal Sciences, University of São Paulo (HOVET/FMVZ-USP), São Paulo, Brazil. The animal belonged to a pre-school located in the municipality of Cotia, state of São Paulo, and was kept in a pasture during the day, along with a horse and three ewes. The goat was fed with alfalfa hay, feed and grazed in the pasture. The goat had a history of ruminal tympany, sternum and limb edema, tachycardia, tachypnea, anorexia, fever, and occasional diarrhea. Its owner reported that the animal had an intermittent dry cough and was treated with an orogastric tube in clinical crisis. However, the animal had several relapses over a few days and was taken to the HOVET/FMVZ-USP, when died during transportation.

**Fig. 1:**
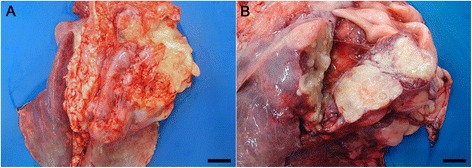
Gross lesions of lungs. **a** The lung was distended and pink with reddish areas and there were multiple nodular areas in right cranial and middle lobes. Bar = 4 cm. **b** Nodular areas containing yellow-whitish gelatinous content. Bar = 2 cm

A complete necropsy was performed, and the macroscopic findings indicated an adequate body condition. No ectoparasites, trauma signs, or marked abdominal distention were present. The gross lesion was restricted to the chest cavity, where 2.0 to 6.0 cm multiple nodular masses were observed in the lungs (Fig. 1a) in cranial and caudal portions of right cranial and in the middle lobes of lung. The nodules were soft and drained a large amount of yellow-whitish gelatinous content at cut (Fig. 1b). Other organs showed no macroscopic alterations.

**Fig. 2:**
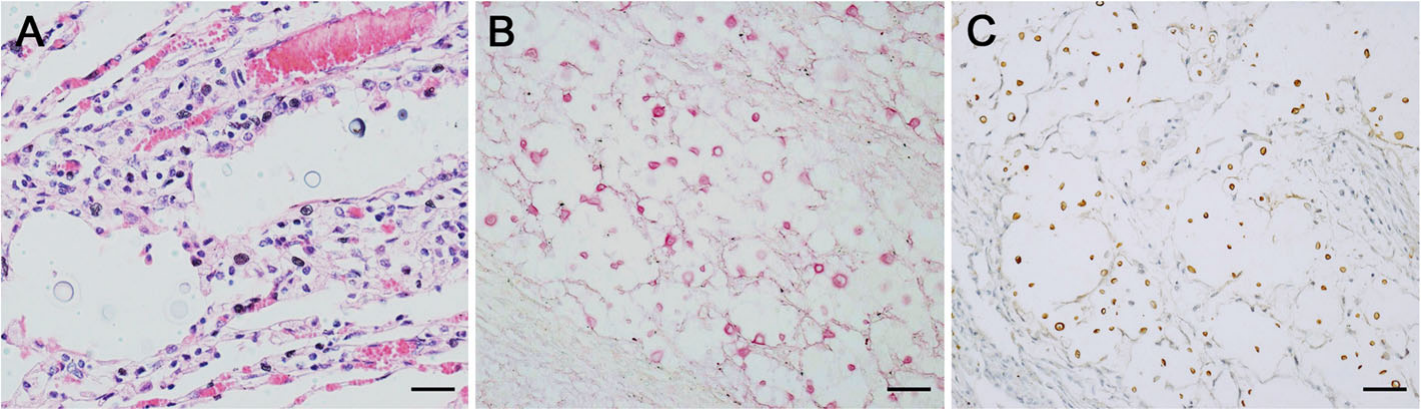
Lung photomicrography. **a** Well-circumscribed cavitations areas of granulomatous pneumonia associated pleomorphic, yeast structures with mucinous capsule of variable thickness which show clear and refractile halo (HE stain, bar = 25 μm). **b** Yeast in details with oval to round, and 5–20 μm in diameter organism’s structure which capsule (carminophilic) stain in red inside of cavitations areas (cryptococcomas). (Mayer’s Mucicarmin stain, bar = 50 μm). **c** Cryptococcal antigen was detected by immunohistochemistry using anti-Cryptococcus polyclonal antibody, 1:50,000 diluted. Positive yeast structures stained in strong brown for *Cryptococcus* spp. PictureMax Kit™, DAB stain, bar = 25 μm

Tissue samples were collected and fixed in 10% buffered formalin and cut in 5 μm sections and stained with hematoxylin-eosin (HE) and Mayer’s Mucicarmin for microscopical analyses. Microscopically, discrete alveolar edema and multifocal, well-circumscribed cavity areas were observed in the lungs. Within these foci there were mild to moderate lymphoplasmacytic and histiocytic inflammatory infiltrate, and rare neutrophils, associated with numerous round extracellular yeasts structures with variable capsular thickness, narrow-based budding, that measured approximately 5.0–20 μm in diameter (Fig. 2a), which stained weakly with HE and strongly stained the capsule by Mayer’s Mucicarmin (Fig. 2b). These areas were characterized of cryptococcomas.

Samples of lung were collected during the necropsy and cultured in Sabouraud-dextrose broth and Sabouraud-dextrose agar with chloramphenicol (100 mg/l), incubated at 25 °C for three days or seven days, respectively, and evaluated daily. Samples grown in Sabouraud-dextrose broth were plated on Sabouraud-dextrose agar with chloramphenicol (100 mg/l), which was incubated as previously described. Beige, mucoid, bright, and viscous colonies with a milky aspect were subjected to morphological evaluation using China ink for capsule visualization. Biochemical tests such as urease, sugar assimilation (glucose, galactose, lactose, melibiose, and sucrose) and potassium nitrate (KNO_3_) tests, as well as evaluation of growth at 37 °C and in media containing cycloheximide, were performed. These tests were performed as described by Lacaz et al. [14] and Larone [15], and their results confirmed identification of the fungal agent as a member of the *Cryptococcus* spp. complex.

Histological sections of lungs were subjected to immunohistochemistry using antibody-specific for *Cryptococcus* spp. Histological sections (3 μm) were subjected to an antigen retrieval protocol in 10 mM citric acid solution (pH 6.0) in a pressure cooker for three minutes at 120 °C. Subsequently, endogenous peroxidase was blocked with H_2_O_2_ for 30 min, followed by non-specific binding blocking (Protein Block - PictureMax Kit™ Life Technologies, Carlsbad, California, USA) for 15 min. The anti-Cryptococcus polyclonal primary *in house* antibody was diluted in phosphate buffered saline (PBS) solution with 1% bovine serum (pH 7.4; 1:50,000), and the slides were incubated with primary antibody overnight at 4 °C. This primary antibody was purified from immunoglobulin fraction of rabbit antiserum, injected with an isolate of *Cryptococcus gatti* (LMM21) serotype B (MAT-alpha), VGII/AFLP6 as described by Kidd et al. [16] and Lourenço [17]. The antibody was tested in immunohistochemistry for reactivity with a variety of organisms. No cross-reactivity was observed with *Histoplasma capsulatum*, Candida, *Pneumocystis carinii*, Cryptosporidia and *Mycobacterium tuberculosis*.

Signal amplification was performed with a PictureMax Kit™ (Life Technologies, Carlsbad, California, USA) for 30 min at 37 °C. Thereafter, the staining was revealed by 100 mg of 3,3′-diaminobenzidine (DAB D-5637; Sigma, St. Louis, Missouri, USA) diluted in PBS (pH 7.4) at 37 °C for three minutes, and counter-staining was performed with Harris’ hematoxylin. Reaction was observed with an optical microscope, which confirmed the presence of structures that were positively stained and compatible with *Cryptococcus* spp. (Fig. 2c).

Genotype of fungus was achieved through PCR and sequencing. Initially, DNA was extracted from frozen lung tissue fragments, stored at -80 °C, according to the manufacturer’s instructions (*Illustra tissue and cells genomic Prep Mini Spin Kit* - *GE Healthcare*, USA). DNA quality and concentration were evaluated by analyzing the integrity and purity of the samples with a NanoDrop (*NanoDrop Technologies*, Wilmington, Delaware, USA).

PCR was performed based on the protocols of Mirhendi et al. [18] and Meyer et al. [19] using the primers ITS1 (5′-TCCGTAGGTGAACCTGCGG-3′) and ITS4 (5′-TCCTCCGCTTATTGATATGC-3′); URA5 (5′-ATGTCCTCCCAAGCCCTCGACTCCG-3′) and SJ01 (5′-TTAAGACCTCTGAACACCGTACTC-3′). Approximately 100 ng of DNA was used for each PCR reaction and FastStart PCR Master (*Roche*, Rotkreuz, Switzerland) in a 25 μl reaction volume. Solution was initially incubated at 94 °C for three minutes for denaturation, followed by 40 cycles of 94 °C for one minute, 58 °C for the first primer pair and 61 °C for the second primer pair for one minute, and 72 °C for one minute, and a final extension of 72 °C for 10 min. Amplicons were visualized on a 2% agarose gel and photographed using an Image Master VDS (*Pharmacia Biotech, USA*). RNase and DNase free ultra-pure water was used as a negative control (UltraPure™DEPC- Treated Water, Invitrogen, USA). PCR product was sequenced by the Center for Human Genome Studies, Institute of Biosciences-USP after purification using a commercial kit, according to the manufacturer’s instructions (*QIAquick PCR Purification Kit, QIAgen*, *Hilden*, Germany). For sequencing, 4 μL of BigDye 3 (*BigDye Terminator v3.1 Cycle Sequencing Kit*, Applied Biosystems, USA), 4 μL of 5X buffer, 0.5 μL of each primer (10 μM ITS1, ITS4, URA5, and SJ01) in separate reactions, 40 ng of target DNA, and RNase- and DNase-free ultra-pure water were used for a final reaction volume of 20 μL in a capillary automated sequencer (*ABIPrism 3730 DNA Analyser*, Applied Biosystems, USA). The quality of the chromatograms generated for each primer (forward and reverse) of each sample was evaluated using the online application Phred (http://asparagin.cenargen.embrapa.br/phph/) and then manually edited using the Chromas Lite v. 2.1.1. software (http://technelysium.com.au/?page_id=13). The final sequence of each sample was obtained using the application Cap-contig of the BioEdit v.5.0.9 software (www.mbio.ncsu.edu/bioedit/bioedit.html) and subjected to a homology search for other sequences deposited in GenBank using the Blast 2.2.29 software (http://www.ncbi.nlm.nih.gov/blast/).

The sequencing results showed 100% homology to a region of the internal transcript sequence 1 (ITS1) of the ribosomal DNA amplified with *C. gattii* strain [GenBank acc # JQ812708 (ATCC MYA-4877)] and 99% homology to IDR1100011626 (GenBank acc # JN675352). Moreover, orotidine monophosphate pyrophosphorylase gene (URA5) signature sequence from this isolate exactly matched that of *C. gattii* corresponds with PCR-fingerprint molecular type VGII.

## Discussion

*C. gattii* is recognized as the main agent of endemic primary cryptococcosis in the North and Northeast (NE) Region of Brazil, where it is responsible for 89% of human cryptococcosis, and has the potential to cause life-threatening disease in immunocompetent hosts and [20]. The first VGII strain (LMM 293) identified in Brazil was isolated in Rio de Janeiro in 1988 from a patient coming from the NE [21]. In 1999, a total of 19 cases of cryptococcosis, nine of them caused by *C. gattii* infections, were described in children in Pará [22]. In the same region, an other study, showed that *C. gattii* is an endemic primary mycosis affecting HIV-negative hosts, with most cases caused by molecular type VGII, including an unexpectedly high number of children, [22, 23].

Most of the clinical isolates of VGII in the states of Rio de Janeiro and São Paulo have been collected from patients born in Northeastern Brazil [24]. In South and Southeast Brazil, 43% of *C. gattii* infections are caused by VGII [21]. However, a diagnosis of meningoencephalitis caused by a VGII strain in a 5-year-old child who has lived all his life in the state of Rio de Janeiro suggests that the VGII strain may be spreading from the Northeast to Southeast region of Brazil. In addition, *C. gattii* has been the agent of one outbreak in captive psittacine birds in São Paulo, Brazil [25].

Also, *C. gattii* molecular type VGII was described as a primary emerging pathogen on Vancouver Island, Canada, and, subsequently, spread to people and animals in British Columbia, Washington and Oregon. Retrospective studies suggested that *C. gattii* may have circulated in Southern California for much longer. The mechanism of the switch from tropical to temperate climates is unknown. However, most eucalyptus associated outbreaks in Australia are of molecular type VGI, whereas 90% of isolates from the Pacific Northwest are type VGIIa, and southern California isolates are type VGIII, the genotype commonly identified in Mexico. This suggests that different genotypes have different biogeoclimatic distributions [26–28].

*C. gattii* can acts as a primary pathogen and causes endemic cryptococcosis, including meningoencephalitis, in HIV-negative patients. However, fully competent immune response in these hosts has been questioned, since infection with *C. gattii* is increasingly linked to the presence of various types of autoantibodies, which interfere with the host defenses, like anti-granulocyte-macrophage colony-stimulating factor [29].

*Cryptococcus* is the most common systemic mycosis in cats and often results in a severe disseminated disease in dogs [30]. According to Torres-Rodríguez et al. [31], the large number of cats and dogs diagnosed with cryptococcosis might be related to evident clinical signs, which facilitate the diagnosis. In other species of domestic animals such as cattle, horses, sheep, and goats, the final diagnosis depends on anatomopathological findings, which hinders the diagnosis. The increased incidence of disease associated with *C. gattii* can be attributed to three factors: environmental expansion, a lack of diagnostic tests, and the increased frequency of identification by molecular detection procedures [32]. Epidemiological studies of cryptococcosis are difficult because fungus species identification is rarely performed [33]. Byrnes et al. [34], emphasize that determination of the molecular type is very important in the clinical and epidemiological clarification of the disease, which highlights the importance of the present study.

Treatment options for cryptococcal infections are dependent on the severity and localization of the infection. In humans, treatment guidelines from the Infectious Diseases Society of America (IDSA) recommend treatment of severe cases of cryptococcosis in immunocompetent and immunocompromised patients using induction therapy for 2 weeks with a combination of amphotericin B and 5-flucytosine, followed by 2 weeks of consolidation therapy using fluconazole [35]. Compared to *C. neoformans*, infections with *C. gattii* might need a more aggressive antifungal therapy due to the higher probability of severe neurological complications and possible delayed response to used antifungal compounds in humans [36]. It has been observed that in vitro antifungal susceptibilities significantly differ between genotype VGI and VGII *C. gattii* strains, which might affect the outcome of antifungal therapy, and which is an indication that in vivo differences may exist [35]. Dose and length of treatment with oral fluconazole for *Cryptococcus* spp. was established on available literature in horses, cats and dogs [37–39]. However, in ruminants, the bioavailability of oral medications usually varies from that in monogastric animals, so intravenous administration has been preferred. One goat with cryptococcal infection in cesarean incision site was treated daily with oral fluconazole for 6 months; however it demonstrated a cryptococcoma peritoneal recurrence after 2 years from the first diagnosis [12].

Despite the few epidemiological studies available in humans and animals, molecular subtype VGII does not seen to be a rare genotype of *C. gattii* in South America. In fact, it behaves as a primary fungal pathogen and causes endemic cryptococcosis in immunocompetent hosts in the northern region of Brazil, where it is particularly well-adapted to environmental biotypes associated with wood decay. However, this molecular type may be adapting to new areas in the southeast due to human activities and global climate change, or it may be present at a low density in the southeast, causing occasional human infections [21].

## Conclusion

Histopathology, immunohistochemistry, microbiology, and molecular test are important to determine infectious agents and their differential diagnosis in cases of systemic respiratory diseases in small ruminants. To our knowledge, this is the first report of a pulmonary infection linked to *C. gattii* molecular type VGII in a goat in state of São Paulo, Brazil. Our findings emphasize the need for an active surveillance program for human and animal infections due to expansion of the epidemiological niche of this important zoonotic microorganism.

## Abbreviations

DAB: 3,3′-diaminobenzidine
DNA: Deoxyribonucleic acid
HE: Hematoxylin-eosin
HOVET/FMVZ-USP: Veterinary Hospital of the School of Veterinary Medicine and Animal Sciences, University of São Paulo
IDSA: Infectious Diseases Society of America
ITS: Internal transcript sequence
KNO_3_: Potassium nitrate
NE: Northeast
PBS: Phosphate buffered saline
PCR: Polymerase chain reaction
URA5: Orotidine monophosphate pyrophosphorylase gene
